# Upregulation of TRIM5α gene expression after live-attenuated simian immunodeficiency virus vaccination in Mauritian cynomolgus macaques, but TRIM5α genotype has no impact on virus acquisition or vaccination outcome

**DOI:** 10.1099/vir.0.047795-0

**Published:** 2013-03

**Authors:** Giada Mattiuzzo, Nicola J. Rose, Neil Almond, Greg J. Towers, Neil Berry

**Affiliations:** 1 Divison of Retrovirology, National Institute for Biological Standards and Control–Health Protection Agency, South Mimms, Potters Bar, Hertfordshire EN6 3QG, UK; 2 Division of Infection and Immunity, University College London, Gower Street, London WC1E 6BT, UK

## Abstract

Polymorphism in the TRIM5α/TRIMcyp gene, which interacts with the lentiviral capsid, has been shown to impact on simian immunodeficiency virus (SIV) replication in certain macaque species. Here, in the context of a live-attenuated SIV vaccine study conducted in Mauritian-origin cynomolgus macaques (MCM), we demonstrate upregulation of TRIM5α expression in multiple lymphoid tissues immediately following vaccination. Despite this, the restricted range of TRIM5α genotypes and lack of TRIMcyp variants had no or only limited impact on the replication kinetics *in vivo* of either the SIVmac viral vaccine or wild-type SIVsmE660 challenge. Additionally, there appeared to be no impact of TRIM5α genotype on the outcome of homologous or heterologous vaccination/challenge studies. The limited spectrum of TRIM5α polymorphism in MCM appears to minimize host bias to provide consistency of replication for SIVmac/SIVsm viruses *in vivo*, and therefore on vaccination and pathogenesis studies conducted in this species.

Simian immunodeficiency virus (SIV) infection in macaques represents a widely used, non-human primate model to study pathogenic lentivirus infection and to evaluate new therapeutic strategies against human immunodeficiency virus (HIV). In live-attenuated SIV vaccination (LAV) studies, significant levels of protection against wild-type virus challenge can be conferred against both homologous (Almond *et al.*, 1995; Berry *et al.*, 2008; Daniel *et al.*, 1992) and heterologous (Berry *et al.*, 2011; Wyand *et al.*, 1999) virus challenge. However, levels of protection vary between different viral challenges and among different host species. Although a live-attenuated HIV vaccine is unlikely ever to be employed due to safety concerns, characterization of the mechanism of protection could unveil novel strategies to reproduce this potent protection safely. In the Mauritian cynomolgus macaque (*Macaca fascicularis*; MCM) model, protection seems to be acting as early as 21 days post-vaccination (Stebbings *et al.*, 2004; Berry *et al.*, 2011), when adaptive responses are either not fully matured or do not appear to be central to the protection observed in this model (Almond *et al.*, 1997; Stebbings *et al.*, 2005).

To extend these studies, we examined whether TRIM5α expression is induced by live-attenuated SIV vaccination and whether TRIM5α polymorphism may play a contributory role in vaccine outcome. TRIM5α is a component of the innate immune system responsible for an intracellular block to retroviruses (Stremlau *et al.*, 2004; Yap *et al.*, 2004), as well as being a sensor for the innate immune response (Pertel *et al.*, 2011). In SIV/macaque studies, polymorphisms in TRIM5α have been correlated with differential control of SIV infection (de Groot *et al.*, 2011; Kirmaier *et al.*, 2010; Lim *et al.*, 2010), suggesting that genotypic variation in TRIM5α and/or expression may impact both on the ability of an attenuated SIV to replicate *in vivo* and, perhaps, on subsequent protection conferred by live-attenuated SIV vaccination. However, expression levels of TRIM5α in tissues susceptible to SIV infection have not been hitherto described. Here, we have measured TRIM5α mRNA levels in a previously reported early-pathogenesis SIV/MCM study (Li *et al.*, 2011). Briefly, 16 MCM were inoculated intravenously with a *nef*-disrupted SIV, SIVmac251/C8, which has been shown to confer protection at 3 and 20 weeks post-infection (Berry *et al.*, 2011, 2008; Stebbings *et al.*, 2004). At these and earlier time points, macaques were sacrificed and multiple lymphoid tissues and blood were collected.

Viral RNA (vRNA) in plasma was detected at day 3, increasing progressively to a peak at day 10; vRNA then declined, but still persisted at low levels at day 125 (Li *et al.*, 2011). Total RNA was isolated from a range of different tissues taken at 0, 3, 7, 10, 21 and 125 days post-inoculation, and TRIM5α and glyceraldehyde-3-phosphate dehydrogenase (GAPDH) RNA levels were quantified by SYBR Green-based quantitative PCR, using primers TRIM5s (5′-CGCTACTGGGTTGATGTGACAC-3′) and TRIM5ns (5′-CCCTGGTGCCTGATACATTATCTG-3′) or GAPDH-s (5′-GGCTGAGAACGGGAAGCTC-3′) and GAPDH-ns (5′-AGGGATCTCGCTCCTGGAA-3′). TRIM5α copy number was normalized to that of GAPDH and expressed as fold difference in comparison to one of the naïve animals (A1; Fig. 1). Despite considerable variation across individual tissues and between vaccinates in response to SIVmac251/C8 infection, there was a significant increase in TRIM5α mRNA expression over days 3, 7 and 10, when all tissues were analysed together and compared with naïve, unvaccinated macaques (*P* = 0.012, two-tailed *t*-test). However, beyond the peak of virus production (day 10), TRIM5α mRNA expression returned to the normal range between days 21 and 125, when the plasma viral RNA levels were low, and the overall virus profile is that of a controlled infection. TRIM5α mRNA kinetics were similar to those observed previously for APOBEC3G in rhesus macaques (Mußil *et al.*, 2011), suggesting a general response to acute retroviral infection, most likely mediated by a type 1 interferon. Whether this increase in restriction factor expression levels influences the antiviral state of the host is difficult to determine, as the transient increase in TRIM5 mRNA levels was not maintained at these higher induction levels beyond the immediate acute phase, and no correlation was found between TRIM5α expression and viral load in plasma (Fig. 1). However, we reasoned that the higher level of TRIM5α expression observed during the peak of primary viraemia could influence subsequent outcome of SIV infection or vaccination, the extent of which could differ in MCM with different TRIM5α genotypes.

**Fig. 1.**
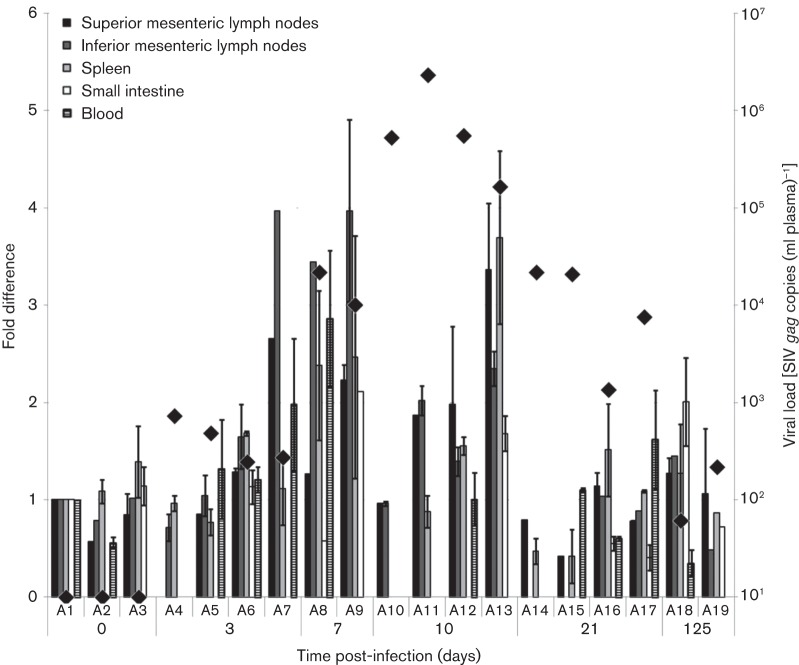
TRIM5α RNA expression level in tissues. Total RNA was extracted from cell-derived tissues and reverse-transcribed, and cDNA equivalent to 20–50 ng total RNA was used in a SYBR Green-based quantitative PCR. PCR product specificity was assessed by dissociation curves. TRIM5α copy numbers were normalized to 5×10^5^ GAPDH copies and the naïve animal A1, used as calibrator. All experiments were run in duplicate and error bars represent the mean±sd of two independent experiments. vRNA in plasma for each animal at the time of termination (⧫) was measured by quantitative RT-PCR as described previously (Berry *et al.*, 2008).

In rhesus macaques (*Macaca mulatta*), variations in the sequence of the TRIM5α B30.2 domain, including its replacement with cyclophilin A, have a great impact on lentiviral infection both *in vivo* and *in vitro* (de Groot *et al.*, 2011; Kirmaier *et al.*, 2010; Lim *et al.*, 2010; Wilson *et al.*, 2008). MCM display limited genetic diversity as a result of a small founder population and geographical isolation (Tosi & Coke, 2007), offering the potential to develop an SIV/macaque model where the confounding effects of host genetics can be minimized. TRIM5α genotypes in cynomolgus macaques of different origin have also been recently characterized (Berry *et al.*, 2012; de Groot *et al.*, 2011; Dietrich *et al.*, 2011; Saito *et al.*, 2012). Only three alleles have been identified in MCM: *mafa-4* (identical to rhesus *mamu-4*) and cynomolgus-specific *mafa-8* and *mafa-9*, but to date no TRIMCyp variants (with cyclophilin A) have been identified (Berry *et al.*, 2012; de Groot *et al.*, 2011; Dietrich *et al.*, 2011). These three alleles, in the B30.2 domain, differ only by three amino acids (M330V and Y389C in *mafa*-8, and I437V in *mafa*-9); however, they all share the Q339TFP polymorphism, which, in rhesus macaques, is associated with a permissive phenotype (Kirmaier *et al.*, 2010; Lim *et al.*, 2010). We extended this genotyping of MCM as described previously (Berry *et al.*, 2012) to a total of 90 MCM. This confirmed the presence of only the three previously identified alleles, with the *mafa-4*/*4* homozygote constituting 56.7 % of the population, and with only four of 90 MCM not carrying the *mafa-4* allele (Fig. 2a).

**Fig. 2.**
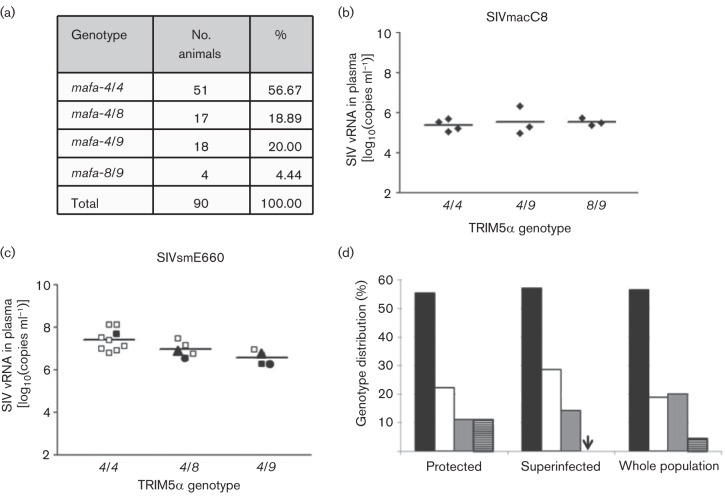
Lack of correlation between SIV infection and TRIM5α genotype. (a) TRIM5α genotype was characterized in 90 MCM. (b, c) Viral load in plasma. MCM were infected intravenously with 5000 TCID_50_ of the 9/90 pool of SIVmacC8 (b), or with 10 (□), 100 (▪), 1000 (▴) or 10 000 (•) MID_50_ of SIVsmE660 (c). Viral load in plasma was determined at 10 (b) or 14 (c) days post-inoculation by quantitative probe-based one-step RT-PCR. Differences between genotypes were not statistically significantly different by one-way ANOVA, Kruskal–Wallis test. The difference between viral loads for genotypes *mafa-4*/*4* versus *mafa-4*/*9* (c) was significant by Dunn’s multiple comparison test (*P*<0.05). (d) MCM were vaccinated with 5×10^3^ TCID_50_ SIVmacC8, and challenged 3 or 20 weeks post-vaccination with 10 MID_50_ of SIVsmE660 or SIVmac251/L28. Six of eight animals challenged with SIVsmE660 and three of eight animals infected with SIVmac251/L28 were protected. One of the two SIVsmE660-superinfected animals and two with SIVmac251/L28 were vaccinated for 3 weeks and the others for 20 weeks. MCM were grouped based on study outcome and compared with the whole population. Percentages of each genotype [*mafa-4*/*4* (black), *-4*/*8* (white), *-4*/*9* (grey) and *-8*/*9* (striped)] were calculated for each group. Distribution of the genotypes among the different groups was not found to be statistically significantly different as assessed by Fisher’s exact test.

We then examined the contribution of each genotype to the level of plasma vRNA at the time of peak viraemia (10–14 days) following intravenous infection with 5000 TCID_50_ of the 9/90 pool of SIVmacC8 (Rud *et al.*, 1994) as used in live-attenuated SIV vaccine studies, or 10–10 000 MID_50_ of an uncloned heterologous SIVsmE660 challenge stock (Berry *et al.*, 2011), representing a wild-type SIVsm-derived virus (Fig. 2b, c). No major differences in viral load at the peak of viraemia for SIVmacC8 could be associated with any of the TRIM5α genotypes (Fig. 2b). Levels of SIVsmE660 in plasma were also similar, regardless of the initial viral dose (Berry *et al.*, 2011) or TRIM5α genotype (Fig. 2c), although the difference in the mean for the *mafa-4*/*4* homozygotes and the *mafa-4*/*9* heterozygotes was significant by Dunn’s multiple comparison test. Hence, there was no strong impact of TRIM5α genotype on acquisition or replication potential of SIVmac or SIVsm *in vivo* in MCM.

In further support of this hypothesis, we retrospectively analysed two previously published LAV vaccination studies. Briefly, 16 MCM were vaccinated with 5×10^3^ TCID_50_ SIVmacC8 and challenged with either SIVmac251/L28 (Berry *et al.*, 2008) or SIVsmE660 (Berry *et al.*, 2011), representing homologous and heterologous challenges, respectively. Taking these two vaccine populations together, irrespective of the composition of the virus challenge, *mafa-4/4* homozygotes constituted 62.5 % of the protected macaques, 50 % of the superinfected and 55.8 % of the total; *mafa-4*/*8* heterozygotes were slightly more represented in the superinfected MCM (28.6 %) than in protected ones or the whole population (18.6 and 18.7 %, respectively). A total of 18.7 % of the protected macaques, 14.3 % of the superinfected and 20 % of all MCM were *mafa-4*/*9* heterozygotes. There was just one macaque with the genotype *mafa-8*/*9*, which was protected from viral rechallenge, but there were insufficient data for statistical analysis. These data suggest that distribution of TRIM5α genotypes among vaccine study populations does not differ significantly between protected and superinfected vaccinated macaques, in comparison with the whole population (Fig. 2d), and hence TRIM5α genotype per se has no impact on vaccine/study outcome. This would appear to hold for both homologous and heterologous virus challenges in such a scenario.

Finally, the three MCM TRIM5α alleles were tested *in vitro* for their ability to restrict lentiviral infection. TRIM5α genes were PCR-cloned using cDNA from animals with genotypes *mafa-4*/*8* and *mafa-4*/*9* and the following primers: sense, 5′-TAGAATTCGCTTCTGGAATCCTGC-3′, and antisense, 5′-TCACGTCGACTCAAGAGCTTGGTGAG-3′ (*Eco*RI and *Sal*I restriction sites underlined). PCR products were subcloned into the gammaretroviral vector EXN (Zhang *et al.*, 2006), downstream and in frame with a haemagglutinin (HA) tag using the restriction enzymes *Eco*RI and *Sal*I. Crandell–Rees feline kidney (CRFK) cells stably expressing TRIM5α alleles were produced as described previously (Ylinen *et al.*, 2010). Similar TRIM5α expression levels were assessed by Western blotting using an anti-HA.11 antibody (Covance; dilution 1 : 1000) and anti-β-actin (Abcam; dilution 1 : 1000), together with an HRP-conjugated anti-mouse IgG antibody (DAKO; 1 : 3000 dilution) (Fig. 3a). TRIM5α-expressing cells were exposed to serial dilutions of VSV-G-pseudotyped, lentiviral vectors derived from HIV-1 (Zufferey *et al.*, 1997), HIV-2 (Griffin *et al.*, 2001) or SIVmac251 (Nègre *et al.*, 2000) carrying a GFP marker gene, and infectious titres were inferred by flow-cytometry analysis. GFP/SIVsmE660 was obtained by replacing SIVmac Gag aa 1–373 with the equivalent residues from SIVsmE660, using *Xho*I and *Age*I restriction sites added to the SIVmac packaging plasmid SIV3+ by mutagenesis. The SIVsmE660 *gag* sequence (GenBank accession no. JX119100) was cloned from vRNA purified from infected animals using the following primers: sense, 5′-TAGAGCTCGAGATGGGCGCGAGAAACTCCGTC-3′, and antisense, 5′-TCGCGACCGGTCTCAGTGCCTCTTTCAATGCTTC-3′ (*Xho*I and *Age*I restriction sites underlined). As expected, HIV-1 vector titre was reduced in cells expressing the simian TRIM5α genes, but no significant reduction of titre was observed for HIV-2, SIVmac251 or SIVsmE660 (Fig. 3b). The non-restrictive phenotype of the MCM TRIM5α alleles, which all carry a glutamine at aa 339, concurs with previous reports (Kirmaier *et al.*, 2010; Lim *et al.*, 2010; Wilson *et al.*, 2008).

**Fig. 3.**
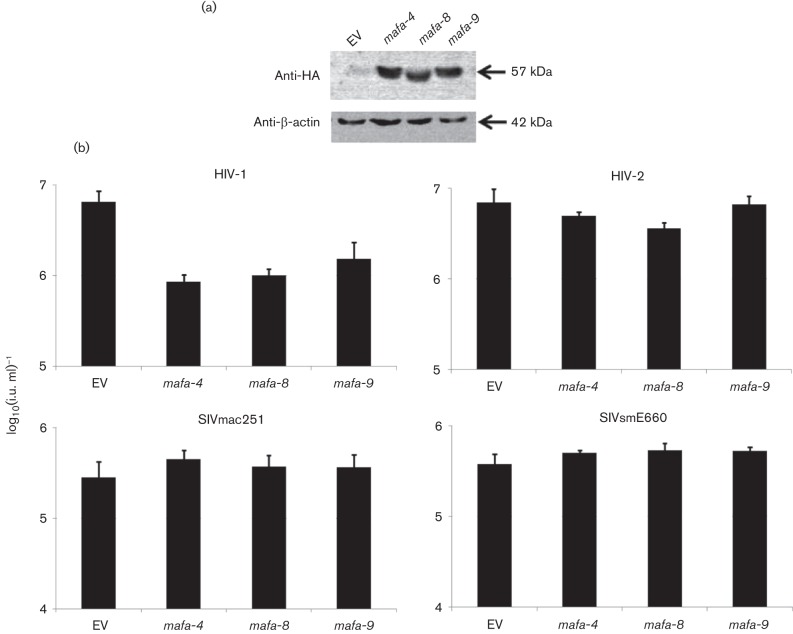
Lentiviral infection in CRFK cells expressing MCM TRIM5α alleles. Feline CRFK cells were transduced using a gammaretroviral vector to express MCM N-terminal HA-tagged TRIM5α alleles *mafa-4*, *mafa-8* and *mafa-9* or an empty vector. (a) Cell lysates were harvested 2 weeks post-TRIM5α expression, after selection with G418, and subjected to immunoblotting using monoclonal anti-HA.11 antibody. β-Actin was detected on the same membrane to assess protein input. (b) CRFK cells were infected with fivefold serial dilutions of GFP-expressing lentiviruses and viral titres were determined by monitoring EGFP expression by flow cytometry. Histograms represent the mean±sem of three independent experiments. Only for HIV-1 was the reduction of viral titre between empty vector (EV) and MCM TRIM5α alleles statistically significant (*t*-test; *P*<0.05).

Although we cannot categorically exclude a contribution of TRIM5α gene expression to the long-term control of SIV/HIV-2 infection *in vivo* and/or vaccination in MCM, we hypothesize that any effects will be minimal, as the only three alleles identified in MCM do not restrict HIV-2, SIVmac251 or SIVsmE660 (Fig. 3). In addition, no correlation between the four different MCM TRIM5α genotypes and outcome of SIV infection in naïve or vaccinated MCM was observed, although a larger number of animals could improve the statistical significance of these observations. MHC genotyping will still be required, as it has been shown that certain MCM haplotypes have an impact on SIV infection in MCM (Mee *et al.*, 2009; Mühl *et al.*, 2002). Animals used in this study have been MHC-genotyped and only two of them (A5 and A17) express allele M6, associated with spontaneous control of infection. No correlation was found with virus replication (Fig. 1).

The results presented here suggest that prerequisite TRIM5α genotyping is of low priority in cynomolgus macaques of Mauritian origin. Our data consolidate the MCM/SIV system as a powerful model to study HIV/AIDS, where bias introduced by host genetics can be reduced to a minimum and rationalized. The replication potential of different SIVmac/SIVsm viruses appears to be largely unimpeded by different TRIM5 genotypes in this non-human primate model of HIV infection and study outcomes unaffected by predisposition to particular TRIM5 variants.
